# Multidisciplinary Antimicrobial Stewardship Program in an Indian ICU: A Time-Series Analysis of Diagnostic Practices and Antibiotic Use

**DOI:** 10.7759/cureus.117062

**Published:** 2026-09-28

**Authors:** Naseema Shaik, Susmitha Simgamsetty, Padmaja Yarlagadda, Uma Penmethsa, Mukesh Kumar Dharmalingam Jothinathan

**Affiliations:** 1 Microbiology, NRI Medical College and General Hospital, Guntur, IND; 2 Microbiology, Saveetha Medical College and Hospital, Chennai, IND; 3 Centre for Global Health Research, Saveetha Medical College and Hospital, Saveetha Institute of Medical and Technical Sciences, Chennai, IND

**Keywords:** antibiotic consumption, antimicrobial resistance, antimicrobial stewardship, de-escalation, diagnostic stewardship, india, intensive care unit, time-series analysis

## Abstract

Background

ICUs are high-risk environments for antimicrobial resistance (AMR). We evaluated the impact of a multidisciplinary Antimicrobial Stewardship Program (AMSP) on diagnostic stewardship, prescribing patterns, and antimicrobial consumption in an Indian tertiary critical care setting.

Methods

A 17-month retrospective observational study was conducted, comparing an eight-month pre-implementation phase (January-August 2024) with a nine-month post-implementation phase (September 2024-May 2025). The AMSP included staff training, structured prescription orders, prospective audits, and monthly feedback. Key metrics included pre-antibiotic culture collection, institutional guideline adherence, surgical antimicrobial prophylaxis (SAP) compliance, and standardized consumption densities (defined daily dose (DDD)/100 bed-days and days of therapy (DOT)/1,000 bed-days), evaluated using interrupted time-series (ITS) regression.

Results

Across 7,682 ICU admissions (3,514 pre- vs. 4,168 post-implementation), pre-antibiotic culture collection increased significantly: blood cultures increased from 331 (20.2%) to 560 (45.8%; p < 0.001), and urine cultures increased from 1,365 (53.0%) to 1,583 (64.6%; p < 0.001). Empirical guideline adherence improved from 1,618 (46.0%) to 3,005 (72.1%; p < 0.001), and SAP compliance increased from 1,352 (35.5%) to 2,083 (63.2%; p < 0.001). Total antimicrobial consumption density decreased by 44.0% (120.04 to 67.17 DDD/100 bed-days; p < 0.001), and duration density declined by 39.6% (981.23 to 592.91 DOT/1,000 bed-days; p < 0.001; ITS regression step-reduction p < 0.001). Meropenem exposure remained strictly controlled and stable (165 (4.7%) vs. 208 (5.0%); p = 0.549).

Conclusion

The multidisciplinary AMSP successfully contained overall antibiotic selection pressure while preserving carbapenems through optimized beta-lactam/beta-lactamase inhibitor utilization. Microbial ecology remained stable, with *Klebsiella* spp. and *Escherichia coli* predominating. Unadjusted case-mix differences represent a recognized study limitation. Implementing a multidisciplinary AMSP achieved sustained reductions in antimicrobial consumption and improved diagnostic practices, providing a feasible framework to combat resistance in resource-limited ICUs.

## Introduction

Antimicrobial resistance (AMR) is a critical global public health threat that jeopardizes the effective treatment of common infections and the safety of modern medical procedures [1]. The ICU represents a focal point for AMR emergence due to the high prevalence of infections, frequent use of broad-spectrum antimicrobials, and the vulnerable patient population [2,3]. Antimicrobial Stewardship Programs (AMSPs) are coordinated interventions designed to promote the judicious use of antimicrobials, thereby improving patient outcomes, reducing adverse events, and curtailing the development of resistance [4,5].

Effective AMSPs employ strategies such as prospective audit and feedback, formulary restrictions, and the development of evidence-based institutional guidelines [6]. Their impact is measured through process indicators (e.g., pre-therapy culture rates), outcome indicators (e.g., adherence to guidelines), and quantitative consumption metrics such as defined daily dose (DDD) per 100 bed-days and days of therapy (DOT) per 1,000 bed-days [7,8].

The Indian subcontinent faces a severe AMR crisis, burdened by high rates of infections caused by multidrug-resistant organisms and complex challenges, including unregulated antibiotic access [9,10]. Although the National Action Plan on AMR (NAP-AMR) advocates for the establishment of AMSPs, their implementation, particularly in critical care settings, remains inconsistent and under-documented [11]. There is a pressing need for robust, localized data demonstrating the feasibility and efficacy of AMSPs within the Indian healthcare context. Given the urgent need to generate evidence on AMSP effectiveness, a retrospective pre-post study design was selected as a robust and pragmatic initial approach. This design allows for the efficient analysis of existing data to evaluate the impact of an AMSP intervention on key prescribing and consumption metrics before investing in a more resource-intensive prospective trial.

This study aimed to evaluate the association of a multidisciplinary AMSP with changes in diagnostic culture practices, prescribing patterns, and antimicrobial consumption in the ICUs of a tertiary care hospital in India. The primary outcomes were antimicrobial consumption measured by DDD per 100 ICU bed-days and DOT per 1,000 ICU bed-days. Secondary outcomes included pre-antibiotic culture collection, empirical guideline adherence, surgical antimicrobial prophylaxis (SAP) compliance, and culture-directed de-escalation.

## Materials and methods

Study design and setting

This study was designed as a retrospective observational study conducted over a 17-month period at the tertiary-level NRI Medical College and General Hospital. The analysis involved patients managed in the Surgical ICU (SICU) and Medical ICU (MICU) from January 2024 through May 2025. Oversight, data collation, and assessment were performed by the hospital’s AMSP committee.

Study phases and intervention

The study was conducted across two distinct chronological phases spanning 17 months. The pre-implementation baseline phase encompassed an eight-month period from January 2024 to August 2024, during which standard ICU care was observed prior to stewardship implementation. The post-implementation phase spanned nine months from September 2024 to May 2025, initiated following the hospital-wide rollout of the multidisciplinary AMSP.

Eligibility criteria, intervention, and data handling

All consecutive adult patients (≥18 years) admitted to the MICU and SICU during the study period who received at least one systemic antimicrobial agent were included. Patients transferred from other facilities with ongoing antimicrobial therapy, those with incomplete prescription records, and those discharged or transferred within 24 hours of ICU admission were excluded. The AMSP was implemented hospital-wide, with the multidisciplinary team conducting prospective audit and feedback rounds three times weekly to review antimicrobial regimens. Clinical and pharmacy records were extracted by two trained infection control nurses using a standardized data extraction form, with any discrepancies verified and resolved by the infection control officer.

Microbiological deduplication excluded repeat isolates of the same species from the same anatomical site within a 14-day window. Longitudinal monthly trends (8 pre- and 9 post-intervention months) were analyzed using interrupted time-series (ITS) segmented linear regression to assess level and slope changes, with autocorrelation evaluated using the Durbin-Watson statistic. The segmented regression model was specified as follows:

\[ Y_t = \beta_0 + \beta_1 T_t + \beta_2 D_t + \beta_3 P_t + \epsilon_t \]

where (Y_t) represents the consumption outcome at month (t); (T_t) represents the time elapsed since the start of the study (months 1-17); (D_t) is a binary indicator for the intervention (0 = pre-implementation and 1 = post-implementation); (P_t) represents the time elapsed since AMSP implementation (0 during the baseline period and 1, 2, ..., 9 during the post-implementation period); (\beta_0) represents the baseline intercept; (\beta_1) represents the baseline secular trend; (\beta_2) represents the immediate level change following implementation; (\beta_3) represents the change in slope after implementation; and (\epsilon_t) represents the error term. First-order autocorrelation was assessed using the Durbin-Watson statistic.

AMSP team composition and roles

The multidisciplinary AMSP team comprised a microbiologist, intensivists, surgeons, anaesthesiologists, and clinical pharmacists.

Hospital Infection Control Officer (Microbiologist)

Compiled the hospital’s antibiogram, revised the antibiotic policy, provided guidance on empirical therapy and SAP protocols, and analyzed antibiotic prescription forms.

ICU Surgeon and Anaesthesiologist 

Interpreted susceptibility data, advised on therapy optimisation (escalation, de-escalation, or intravenous-to-oral switch), and monitored antimicrobial usage and dosage accuracy.

Clinical Pharmacists

Calculated antimicrobial consumption using the metrics DDD per 100 bed-days and DOT per 1,000 bed-days.

Data collection and implementation process

During the initial phase, healthcare staff received targeted instruction on the appropriate use and completion of an antibiotic prescription form. Required entries included the patient’s prior exposure to antimicrobial drugs, estimated glomerular filtration rate (eGFR), timing of specimen collection in relation to the start of antibiotics (pre-antibiotic specimen collection), selection of initial (empirical) therapy aligned with department-specific protocols, including SAP measures, and therapy adjustments according to culture and sensitivity results.

For pre-antibiotic specimen collection, a culture was defined as pre-antibiotic if collected before the first dose of systemic antimicrobial therapy, determined by cross-referencing the collection timestamp with the antibiotic start time. Specimens collected within 30 minutes after antibiotic initiation were excluded to account for documentation delays.

Following AMSP implementation, prescription card completion became routine. Monthly retrospective evaluation of the collected data was undertaken to monitor compliance and outcomes.

Microbiological methods

Blood culture specimens were processed using an automated detection system (BACT/ALERT®; bioMérieux, France). Organism identification and antibiotic susceptibility testing were performed using the VITEK 2 Compact system (bioMérieux, France) in accordance with Clinical and Laboratory Standards Institute (CLSI) guidelines [12]. Aggregated microbiology results were compiled into periodic hospital antibiograms, which served as the foundation for updating the institutional antimicrobial policy.

Outcome indicators

The study evaluated two distinct categories of stewardship outcome measures: clinical process indicators of policy adherence and quantitative metrics of population-level antimicrobial exposure.

Clinical and diagnostic policy adherence was assessed across three core operational parameters: the pre-antibiotic diagnostic culture collection rate, the culture-directed de-escalation rate, and the SAP adherence rate. Pre-antibiotic diagnostic sampling was defined as the proportion of cultures collected prior to administration of the initial dose of systemic antibacterial therapy, calculated independently for blood, urine, exudate, lower respiratory tract, and sterile body fluid specimens. To prevent confounding by prior antimicrobial exposure, microbiological diagnostic yield (culture positivity) was calculated exclusively within the pre-antibiotic culture subset. Culture-directed de-escalation adherence was evaluated as the proportion of culture-positive episodes in which spectrum narrowing or discontinuation was performed relative to all clinically eligible de-escalation opportunities. Compliance with institutional SAP protocols was assessed by auditing the selection of indicated narrow-spectrum agents, administration within 60 minutes prior to surgical incision, and cessation within 24 hours postoperatively.

$\text{SAP Adherence Rate (\%)} = \left( \frac{\text{Number of surgical procedures with guideline-compliant prophylaxis}}{\text{Total number of audited surgical procedures}} \right) \times 100$

$\text{DDD per 100 Bed-Days} = \frac{\left( \frac{\text{Total grams of antimicrobial agent consumed}}{\text{WHO-defined DDD in grams}} \right)}{\text{Total occupied ICU bed-days}} \times 100$

$\text{DOT per 1,000 Bed-Days} = \left( \frac{\text{Total days of therapy administered for each antimicrobial agent}}{\text{Total occupied ICU bed-days}} \right) \times 1000$

Quantitative antimicrobial consumption was evaluated using two standardized pharmaco-epidemiological metrics: DDD per 100 occupied bed-days and DOT per 1,000 bed-days. Metrics were determined in accordance with the WHO Collaborating Centre for Drug Statistics Methodology ATC/DDD 2025 index and the CDC National Healthcare Safety Network (NHSN) definitions [13]. The cumulative number of DDDs for each agent was calculated by dividing the total grams of the drug administered by the WHO-established standard adult daily maintenance dose. Patient case sheets and medication administration records were audited to compute total days of therapy, where any administration of a systemic antimicrobial agent on a calendar day represented one DOT, regardless of dose frequency. Both metrics were normalized against total occupied ICU bed-days during the corresponding observation period.

Statistical analysis

Continuous variables (age, monthly DDD/100 bed-days, and DOT/1,000 bed-days) were expressed as mean ± SD and compared using independent two-tailed Student’s t-tests (df = 15 for monthly population metrics). Categorical variables were evaluated using Pearson’s chi-square (χ²) test (df = 1, or df = k - 1 for multi-category distributions), with absolute risk differences reported alongside 95% CI. The Benjamini-Hochberg false discovery rate procedure was applied to adjust for multiple secondary drug comparisons (q < 0.05). Longitudinal 17-month trends (8 pre- and 9 post-intervention points) were analyzed using ITS segmented linear regression to quantify immediate level (β2) and slope (β3) changes. Statistical significance was set at a two-tailed p < 0.05. Analyses were conducted using SPSS version 28.0 and R version 4.3.1.

## Results

Patient demographics

A total of 7,682 ICU admissions were analyzed across the 17-month study period (3,514 (45.7%) pre-implementation and 4,168 (54.3%) post-implementation); baseline patient age remained comparable across cohorts (mean 57.2 ± 13.8 vs. 57.8 ± 13.2 years; p = 0.052). The post-implementation cohort demonstrated significant demographic shifts, characterized by an increase in SICU admissions from 1,684 (47.9%) to 2,333 (56.0%; p < 0.001) and a higher proportion of male patients from 1,970 (56.1%) to 2,586 (62.0%; p < 0.001). The absolute volume of SICU admissions increased by 38.5% (649 additional admissions; from 1,684 to 2,333). This increase in SICU patient volume reflected a higher surgical operative volume during the post-implementation period, while the absolute number of MICU admissions remained stable (1,830 (52.1%) vs. 1,835 (44.0%)). Baseline demographic parameters and comparative statistics are summarized in Table 1.

**Table 1:** Patient demographics pre- and post-AMSP implementation *Categorical comparisons were evaluated using Pearson’s chi-square (χ²) test (df = 1); the continuous age comparison was evaluated using an independent-samples Student’s t-test (df = 7,680). **Mean difference in years. AMSP: Antimicrobial Stewardship Program; MICU: Medical Intensive Care Unit; SICU: Surgical Intensive Care Unit.

Criteria	Pre-implementation phase (N = 3,514)	Post-implementation phase (N = 4,168)	Difference (95% CI)	Test statistic (df)	p-value
Unit type, n (%)					
MICU	1,830 (52.1%)	1,835 (44.0%)	-8.1% (-10.3% to -5.8%)	χ² = 49.54 (1)	<0.001
SICU	1,684 (47.9%)	2,333 (56.0%)	+8.1% (+5.8% to +10.3%)		
Sex, n (%)					
Male	1,970 (56.1%)	2,586 (62.0%)	+6.0% (+3.8% to +8.2%)	χ² = 28.28 (1)	<0.001
Female	1,544 (43.9%)	1,582 (38.0%)	-6.0% (-8.2% to -3.8%)		
Male-to-female ratio	1.28:1	1.63:1	-	-	-
Age (years)					
Mean ± SD	57.2 ± 13.8	57.8 ± 13.2	+0.60 (-0.01 to +1.21)**	t = 1.94 (7,680)	0.052
Range	18-95	18-95	-	-	-

Diagnostic culture practices

Following AMSP implementation, a marked overall improvement in pre-antibiotic culture collection was observed across the ICU, rising from 3,241 (42.3%) of 7,663 specimens to 3,767 (58.5%) of 6,439 specimens submitted (p < 0.001). Pre-antibiotic collection rates increased significantly across lower respiratory tract specimens (from 564 (34.4%) of 1,638 to 720 (70.0%) of 1,029), blood (from 331 (20.2%) of 1,635 to 560 (45.9%) of 1,221), exudates (from 914 (58.6%) of 1,560 to 870 (67.7%) of 1,286), and urine (from 1,365 (53.0%) of 2,576 to 1,583 (64.6%) of 2,450; p < 0.001 for all four anatomical sites). Sterile body fluid cultures were the sole exception, for which pre-antibiotic collection decreased from 67 (26.4%) of 254 to 34 (7.5%) of 453 (p < 0.001).

​Evaluating diagnostic yield among samples drawn prior to antimicrobial exposure revealed distinct site-specific patterns. Blood culture positivity remained virtually unchanged between phases (106 (32.0%) of 331 pre-implementation vs. 174 (31.1%) of 560 post-implementation; p = 0.764). In contrast, diagnostic yields increased significantly for urine (from 347 (25.4%) of 1,365 to 558 (35.2%) of 1,583; p < 0.001) and exudates (from 582 (63.7%) of 914 to 677 (77.8%) of 870; p < 0.001). Respiratory cultures showed a clear decline in positivity from 472 (83.7%) of 564 to 449 (62.4%) of 720 (p < 0.001), reflecting tighter clinical filtering of airway colonizers. No bacterial growth was recovered from sterile body fluids during either period (0 (0.0%)). Across all pre-antibiotic specimens combined, culture yield increased modestly from 1,507 (46.5%) of 3,241 to 1,858 (49.3%) of 3,767 (p = 0.019). Site-specific metrics and comparative test statistics are detailed in Table 2.

**Table 2:** Site-specific diagnostic culture practices and yield pre- and post-AMSP implementation. *Proportions were compared using Pearson’s chi-square (χ²) test (df = 1). Specimen denominators include multiple specimens per patient. Positivity denominators represent the pre-antibiotic culture subset. Abx: Antibiotic; LRTI: Lower respiratory tract infection; NA: Not applicable; AMSP: Antimicrobial Stewardship Program.

Specimen	Measure	Pre-implementation phase, n/N (%)	Post-implementation phase, n/N (%)	Absolute risk difference (95% CI)	χ² (df = 1)	p-value
Blood	Pre-Abx specimen collection	331/1,635 (20.2%)	560/1,221 (45.9%)	+25.6% (+22.2% to +29.0%)	210.5	<0.001
	Positivity	106/331 (32.0%)	174/560 (31.1%)	-1.0% (-7.3% to +5.4%)	0.09	0.764
Urine	Pre-Abx specimen collection	1,365/2,576 (53.0%)	1,583/2,450 (64.6%)	+11.6% (+8.9% to +14.3%)	70	<0.001
	Positivity	347/1,365 (25.4%)	558/1,583 (35.2%)	+9.8% (+6.5% to +13.1%)	33.5	<0.001
Exudates	Pre-Abx specimen collection	914/1,560 (58.6%)	870/1,286 (67.7%)	+9.1% (+5.5% to +12.6%)	25.3	<0.001
	Positivity	582/914 (63.7%)	677/870 (77.8%)	+14.1% (+10.0% to +18.3%)	42.7	<0.001
LRTI	Pre-Abx specimen collection	564/1,638 (34.4%)	720/1,029 (70.0%)	+35.5% (+31.9% to +39.2%)	328.7	<0.001
	Positivity	472/564 (83.7%)	449/720 (62.4%)	-21.3% (-26.0% to -16.7%)	70.7	<0.001
Body fluids	Pre-Abx specimen collection	67/254 (26.4%)	34/453 (7.5%)	-18.9% (-24.8% to -12.9%)	47.7	<0.001
	Positivity	0/67 (0.0%)	0/34 (0.0%)	-	-	NA
Total	Pre-Abx specimen collection	3,241/7,663 (42.3%)	3,767/6,439 (58.5%)	+16.2% (+14.6% to +17.8%)	370.5	<0.001
	Positivity	1,507/3,241 (46.5%)	1,858/3,767 (49.3%)	+2.8% (+0.5% to +5.2%)	5.53	0.019

Pathogen distribution

A total of 3,365 non-duplicate bacterial pathogens were recovered from pre-antibiotic cultures over the study duration: 1,507 in the baseline phase and 1,858 following AMSP implementation. Gram-negative bacilli dominated the infection spectrum during both periods. *Klebsiella spp. *were the most frequent isolates (655 (43.5%) of 1,507 pre- vs. 828 (44.6%) of 1,858 post-implementation; p = 0.528), followed closely by *Escherichia coli *(617 (40.9%) vs. 794 (42.7%); p = 0.291). Among non-fermenters, *Acinetobacter spp.* recovery remained largely unchanged (162 (10.8%) vs. 181 (9.7%); p = 0.344), whereas *Pseudomonas spp.* demonstrated a significant relative decline post-intervention, from 44 (2.9%) to 25 (1.3%; p < 0.001). Other Gram-positive organisms accounted for a small fraction of culture-confirmed infections, with *Staphylococcus aureus* and other Gram-positive cocci remaining stable at low levels (29 (1.9%) vs. 30 (1.6%); p = 0.506). Overall, the cumulative pathogen spectrum exhibited a statistically significant shift between the two phases (χ² = 12.39, df = 4, p = 0.015; Table 3).

**Table 3:** Distribution of bacterial pathogens isolated from pre-antibiotic ICU cultures pre- and post-AMSP implementation. *Proportions and the overall distribution were compared using Pearson’s chi-square (χ²) test. GPC: Gram-positive cocci.

Pathogen isolated	Pre-implementation phase, n (%)	Post-implementation phase, n (%)	Absolute risk difference (95% CI)	χ² test (df = 1)	p-value
Klebsiella spp.	655 (43.5%)	828 (44.6%)	+1.1% (-2.3% to +4.5%)	0.4	0.528
Escherichia coli	617 (40.9%)	794 (42.7%)	+1.8% (-1.5% to +5.1%)	1.11	0.291
Acinetobacter spp.	162 (10.8%)	181 (9.7%)	-1.1% (-3.1% to +0.9%)	0.9	0.344
Pseudomonas spp.	44 (2.9%)	25 (1.3%)	-1.6% (-2.6% to -0.6%)	10.98	<0.001
*Staphylococcus aureus* / other GPC	29 (1.9%)	30 (1.6%)	-0.3% (-1.2% to +0.6%)	0.44	0.506
Total	1,507 (100%)	1,858 (100%)	-	12.39 (df = 4)	0.015

Antimicrobial stewardship interventions

Following AMSP implementation, substantial improvements were observed across clinical decision-making metrics and institutional protocol adherence. Among culture-positive episodes (N = 1,507 pre-implementation vs. N = 1,858 post-implementation), susceptibility-guided antibiotic escalation increased significantly from 362 (24.0%) to 985 (53.0%; χ² = 294.50, df = 1, p < 0.001), ensuring prompt, targeted coverage for resistant pathogens. Culture-directed de-escalation accounted for 858 (56.9%) of baseline episodes compared with 402 (21.6%) post-implementation (χ² = 443.80, df = 1, p < 0.001), while appropriate continuation of empirical regimens without modification increased from 287 (19.0%) to 471 (25.3%; χ² = 19.40, df = 1, p < 0.001).

Across the entire ICU population, adherence to institutional empirical prescribing guidelines improved from 1,618 (46.0%) of 3,514 to 3,005 (72.1%) of 4,168 (χ² = 538.20, df = 1, p < 0.001). Appropriate continuation of empirical therapy in culture-negative or clinically stable patients similarly increased from 1,352 (38.5%) of 3,514 to 2,783 (66.8%) of 4,168 (χ² = 614.90, df = 1, p < 0.001). Compliance with institutional SAP guidelines increased significantly from 1,352 (35.5%) of 3,812 eligible procedures during the pre-implementation phase to 2,083 (63.2%) of 3,296 procedures post-implementation (χ² = 544.00, df = 1, p < 0.001). Improvements were primarily driven by optimizing preoperative timing within 60 minutes prior to surgical incision and eliminating unjustified postoperative continuation beyond 24 hours. Intervention metrics and comparative statistics are summarized in Table 4.

**Table 4:** Antimicrobial stewardship interventions, guideline adherence, and surgical prophylaxis compliance. *Evaluated using Pearson’s chi-square (χ²) test (df = 1). SAP: Surgical Antimicrobial Prophylaxis.

Stewardship metric	Pre-implementation phase, n (%)	Post-implementation phase, n (%)	Absolute risk difference (95% CI)	χ² test (df = 1)	p-value
a. Culture-directed interventions (N = 1,507 pre vs. N = 1,858 post)					
Escalation	362 (24.0%)	985 (53.0%)	+29.0% (+25.8% to +32.2%)	294.5	<0.001
De-escalation	858 (56.9%)	402 (21.6%)	-35.3% (-38.5% to -32.1%)	443.8	<0.001
No change/continued therapy	287 (19.0%)	471 (25.3%)	+6.3% (+3.5% to +9.1%)	19.40	<0.001
b. Institutional policy adherence (N = 3,514 pre vs. N = 4,168 post)					
Empirical therapy continuation	1,352 (38.5%)	2,783 (66.8%)	+28.3% (+26.1% to +30.5%)	614.9	<0.001
Overall policy adherence rate	1,618 (46.0%)	3,005 (72.1%)	+26.1% (+24.0% to +28.2%)	538.2	<0.001
c. SAP (N = 3,812 pre vs. N = 3,296 post)					
SAP compliance	1,352 (35.5%)	2,083 (63.2%)	+27.7% (+25.5% to +29.9%)	544	<0.001

Antimicrobial prescribing exposure and population consumption dynamics

Prescribing exposure was evaluated alongside overall consumption metrics across all systemic antibacterial agents (WHO ATC J01 class) [13]. Because ICU patients frequently received combination coverage or sequential line switches, patient-level exposure categories were non-mutually exclusive, resulting in cumulative totals exceeding 100%.

Carbapenem prescribing remained strictly restricted throughout both periods. Meropenem exposure was low and stable (165 of 3,514 (4.7%) pre-implementation vs. 208 of 4,168 (5.0%) post-implementation; p = 0.549), reflecting post-implementation consumption of just 0.92 DDD/100 bed-days and 18.41 DOT/1,000 bed-days. This stable, low baseline aligns with pre-authorization guidelines reserving carbapenems for severe septic shock or confirmed multidrug-resistant pathogens. Conversely, significant increases were observed in patient exposure to carbapenem-sparing β-lactam/β-lactamase inhibitor combinations, including piperacillin-tazobactam (from 808 (23.0%) to 1,184 (28.4%); p < 0.001) and cefoperazone-sulbactam (from 1,581 (45.0%) to 2,030 (48.7%); p = 0.001). Ciprofloxacin exposure dropped significantly from 1,627 (46.3%) to 1,834 (44.0%; p = 0.044), with its duration density falling from 33.70 to 19.50 DOT/1,000 bed-days. Prescribing rates for oral step-down and targeted options, such as amoxicillin-clavulanic acid (from 1,065 (30.3%) to 1,500 (36.0%); p < 0.001), metronidazole (from 1,019 (29.0%) to 1,355 (32.5%); p = 0.001), cotrimoxazole (from 422 (12.0%) to 609 (14.6%); p = 0.001), and cefuroxime (from 562 (16.0%) to 759 (18.2%); p = 0.010), rose modestly, reflecting active stewardship efforts promoting timely IV-to-oral conversion. Ceftriaxone exposure shifted from 1,553 (44.2%) to 1,930 (46.3%; p = 0.064), while other antimicrobial agents accounted for 1,230 (35.0%) and 1,430 (34.3%; p = 0.524).

At the population level, longitudinal monthly tracking showed a clear, sustained drop in total drug pressure following AMSP initiation at Month 9. Cumulative antimicrobial consumption density fell by 44.0%, from a baseline mean of 120.04 DDD/100 bed-days (range: 108.70-132.70) to 67.17 DDD/100 bed-days post-intervention (p < 0.001; Figure 1). Total therapeutic duration density similarly decreased by 39.6%, from a baseline mean of 981.23 DOT/1,000 bed-days (range: 910.00-1,032.50) to 592.91 DOT/1,000 bed-days (range: 505.00-720.40; p < 0.001; Figure 2). Individual drug metrics and exposure comparisons are summarized in Table 5.

**Figure 1:**
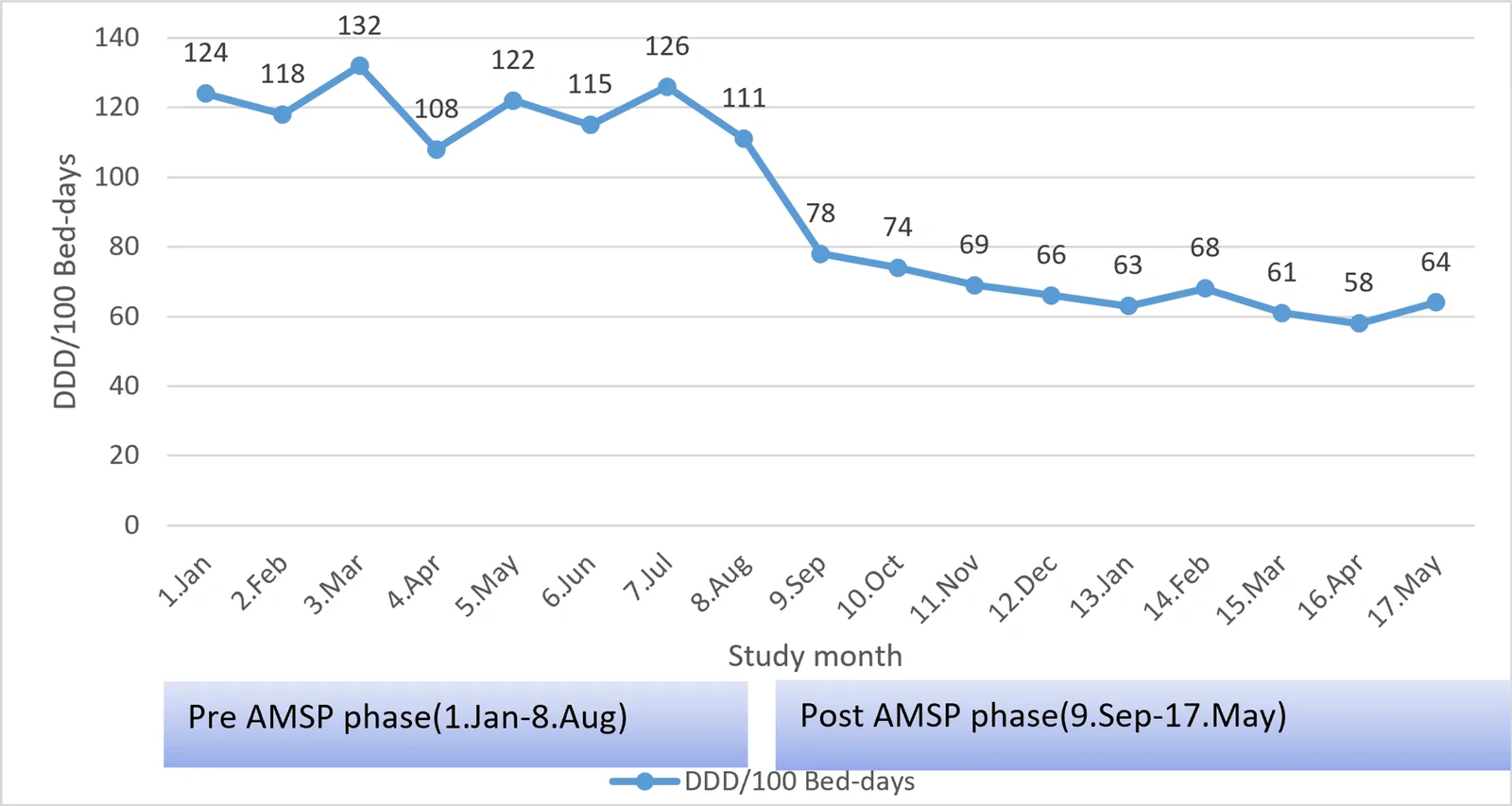
Monthly trend in total antimicrobial consumption density (DDD per 100 bed-days) across the 17-month study period (January 2024 to May 2025). DDD: Defined Daily Dose; AMSP: Antimicrobial Stewardship Program.

**Figure 2:**
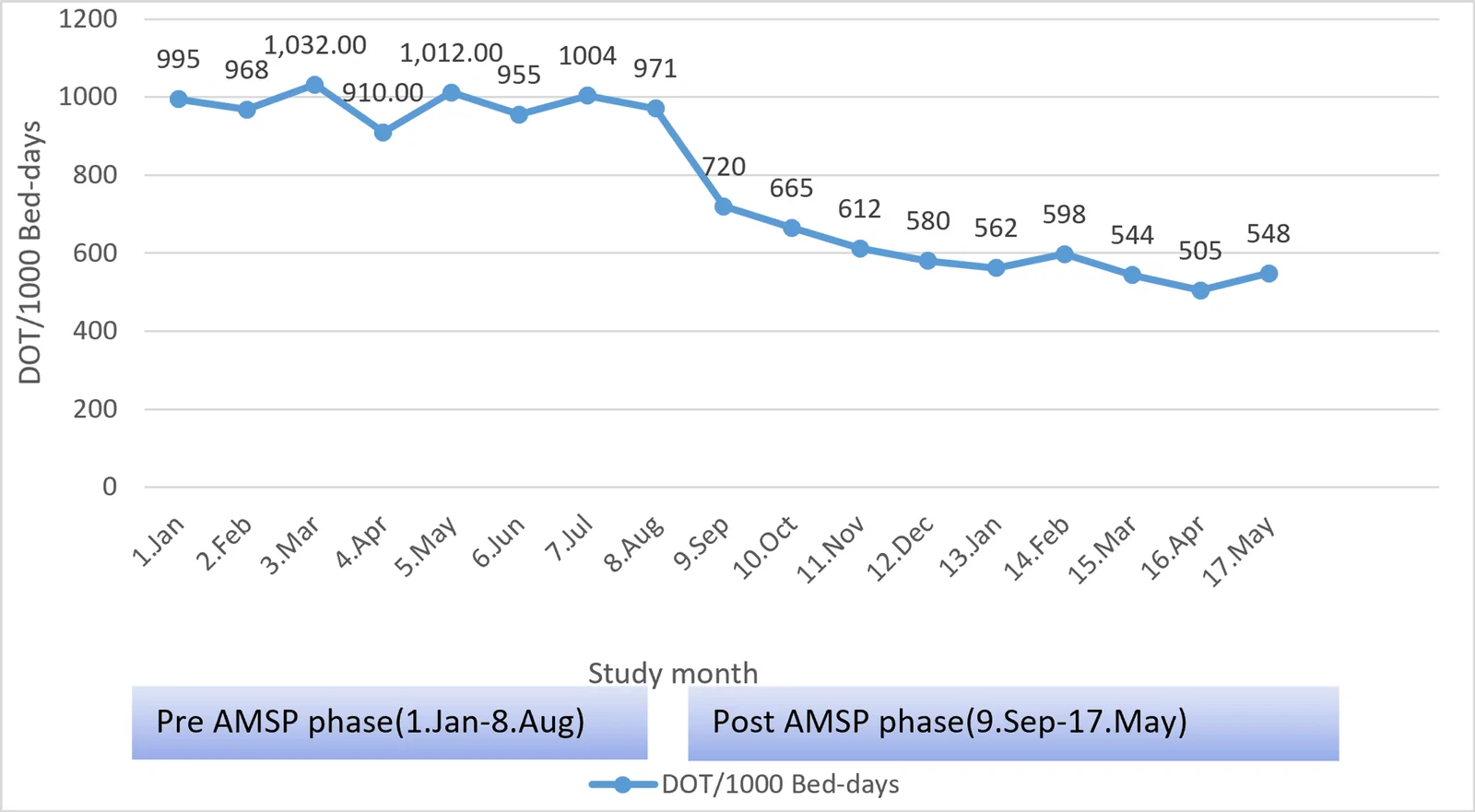
Monthly trend in total antimicrobial therapeutic duration density (DOT per 1,000 bed-days) across the 17-month study period (January 2024 to May 2025). AMSP: Antimicrobial Stewardship Program; DOT: Days of Therapy.

**Table 5:** Individual antimicrobial agent prescribing exposure prevalence, population consumption density (DDD/100 bed-days), and duration density (DOT/1,000 bed-days) pre- and post-AMSP implementation. *Evaluated using Pearson’s chi-square (χ²) test (df = 1). Patient exposure categories are non-mutually exclusive due to concurrent combination regimens and sequential switches; therefore, totals exceed 100%. DDD: Defined Daily Dose; DOT: Days of Therapy.

Antimicrobial agent	Pre-exposure, n (%) (N = 3,514)	Post-exposure, n (%) (N = 4,168)	Absolute risk difference (95% CI)	χ² (df = 1)	p-value	DDD/100 bed-days, pre	DDD/100 bed-days, post	DOT/1,000 bed-days, pre	DOT/1,000 bed-days, post
Meropenem	165 (4.7%)	208 (5.0%)	+0.3% (-0.7% to +1.3%)	0.36	0.549	1.91	0.92	38.3	18.41
Cotrimoxazole	422 (12.0%)	609 (14.6%)	+2.6% (+1.1% to +4.1%)	11.11	0.001	1.15	1.45	18.5	23.2
Cefuroxime	562 (16.0%)	759 (18.2%)	+2.2% (+0.5% to +3.9%)	6.58	0.01	3.85	2.38	38.46	23.83
Piperacillin-tazobactam	808 (23.0%)	1,184 (28.4%)	+5.4% (+3.5% to +7.3%)	29.09	<0.001	0.88	1.56	12.25	21.66
Metronidazole	1,019 (29.0%)	1,355 (32.5%)	+3.5% (+1.4% to +5.6%)	11.01	0.001	6.38	6.94	95.7	104
Amoxicillin-clavulanic acid	1,065 (30.3%)	1,500 (36.0%)	+5.7% (+3.6% to +7.8%)	27.67	<0.001	17.52	11.27	175.2	112.7
Ceftriaxone	1,553 (44.2%)	1,930 (46.3%)	+2.1% (-0.1% to +4.3%)	3.43	0.064	4.2	3.79	84	75.85
Cefoperazone-sulbactam	1,581 (45.0%)	2,030 (48.7%)	+3.7% (+1.5% to +5.9%)	10.55	0.001	1.65	0.98	66.12	39
Ciprofloxacin	1,627 (46.3%)	1,834 (44.0%)	-2.3% (-4.5% to -0.1%)	4.07	0.044	3.37	1.95	33.7	19.5
Others	1,230 (35.0%)	1,430 (34.3%)	-0.7% (-2.8% to +1.4%)	0.41	0.524	79.13	35.93	419	154.76
Total	-	-	-	-	<0.001*	120.04	67.17	981.23	592.91

Monthly antimicrobial consumption densities were tracked across the pre-implementation baseline phase (Months 1-8; January to August 2024) and post-implementation phase (Months 9-17; September 2024 to May 2025) following the initiation of AMSP in September 2024. The pre-implementation baseline phase demonstrated a mean consumption density of 120.04 DDD/100 bed-days (range: 108.70-132.70), whereas the post-implementation phase exhibited a significant 44.0% reduction to a mean of 67.17 DDD/100 bed-days (range: 58.20-78.40; Student’s t-test, p < 0.001).

Monthly therapeutic duration densities were tracked across the pre-implementation baseline phase (Months 1-8; January to August 2024) and post-implementation phase (Months 9-17; September 2024 to May 2025) following the initiation of AMSP in September 2024. The pre-implementation baseline phase demonstrated a mean duration density of 981.23 DOT/1,000 bed-days (range: 910.00-1,032.50), whereas the post-implementation phase exhibited a significant 39.6% reduction to a mean of 592.91 DOT/1,000 bed-days (range: 505.00-720.40; Student’s t-test, p < 0.001).

## Discussion

This retrospective pre-post observational study demonstrates a significant positive association between the implementation of a multidisciplinary AMSP and improved antimicrobial utilization practices in an Indian ICU setting [14-16].

Following stewardship rollout, we observed substantial gains in diagnostic culture collection prior to antibiotic initiation, improved adherence to institutional prescribing guidelines, and marked reductions in overall population antimicrobial exposure and duration density [17,18]. These findings align with recent evidence on hospital stewardship effectiveness across South Asian healthcare systems while providing crucial, context-specific data from a resource-limited tertiary environment where high baseline AMR rates intersect with evolving stewardship infrastructure [14,15,18]. Interpretation of our findings must account for the baseline demographic and case-mix shifts observed between study phases [3, 14]. While mean patient age remained comparable across cohorts (57.2 ± 13.8 vs. 57.8 ± 13.2 years; p = 0.052), the post-implementation period saw a significant increase in SICU admissions (56.0% vs. 47.9%; p < 0.001) and male representation (62.0% vs. 56.1%; p < 0.001), shifting the male-to-female ratio from 1.28:1 to 1.63:1.

The increase in SICU admissions was 38.5%, reflecting increased surgical cases during the post-implementation phase. In an uncontrolled pre-post design, unadjusted case-mix differences represent a recognized threat to internal validity, as surgical and medical critical care cohorts differ fundamentally in baseline infection risks, empirical broad-spectrum requirements, and perioperative prophylaxis protocols [18,19]. Because routine illness severity scores (such as APACHE II or SOFA) and individual clinical endpoints were unavailable in this operational registry, multivariate adjustment could not be performed, representing an important methodological limitation common to real-world stewardship evaluations in resource-constrained centres [18,20].

Enhancing diagnostic stewardship was a central operational objective of the multidisciplinary AMSP, reflected in a significant overall increase in pre-antibiotic culture collection (58.5% vs. 42.3%; p < 0.001) [17, 20]. Securing microbiological specimens prior to antimicrobial exposure is fundamental to maximizing pathogen recovery, preventing culture-negative sepsis misclassification, and enabling targeted pathogen-directed therapy [20, 21].

Site-specific yield analyses revealed distinct microbiological patterns. Blood culture positivity remained stable across phases (31.1% post-implementation vs. 32.0% pre-implementation; p = 0.764), matching standard bacteraemia detection baselines reported in Indian critical care settings [15,21]. In contrast, diagnostic yields increased significantly for urine (35.2% vs. 25.4%; p < 0.001) and exudates (77.8% vs. 63.7%; p < 0.001). Because all analysed specimens were obtained prior to antimicrobial initiation, this increased diagnostic yield is associated with improved pre-analytical specimen collection techniques, higher compliance with aseptic aspirate/tissue sampling over superficial wound swabbing, and enhanced clinician education regarding appropriate clinical thresholds for culturing symptomatic ICU patients. Positivity among lower respiratory tract specimens declined from 83.7% to 62.4% (p < 0.001). In mechanically ventilated ICU patients, elevated baseline respiratory culture positivity frequently represents routine culturing of endotracheal colonizers rather than true parenchymal infection; this reduction points to improved clinical filtering following stewardship education that restricted sputum and endotracheal aspirate sampling to patients exhibiting objective clinical, inflammatory, and radiological signs of active pneumonia [22,23]. Sterile body fluid cultures yielded no bacterial growth across both periods (0.0%), reflecting strict aseptic sampling alongside low baseline pre-antibiotic fluid collection volumes [20].

Gram-negative bacilli dominated the microbial spectrum across both study phases, reflecting the typical epidemiological profile observed in Indian critical care units [15,23]. *Klebsiella spp.* were the predominant pathogens recovered (43.5% pre- vs. 44.6% post-implementation; p = 0.528), followed closely by *Escherichia coli* (40.9% vs. 42.7%; p = 0.291), demonstrating stable baseline colonization and infection dynamics within the ICU environment [15,24]. Among non-fermenting Gram-negative bacilli, *Acinetobacter spp.* isolates remained virtually unchanged (10.8% vs. 9.7%; p = 0.344), whereas *Pseudomonas spp.* demonstrated a significant relative decline post-intervention (2.9% vs. 1.3%; p < 0.001). This reduction in *Pseudomonas spp.* recovery aligns with stewardship-driven rationalization of antipseudomonal agents and improved device-care bundles [25]. Gram-positive cocci accounted for a minor fraction of culture-confirmed infections, with *Staphylococcus aureus *remaining stable at low levels (1.9% vs. 1.6%; p = 0.506). Although the cumulative distribution showed a statistically significant shift (χ² = 12.39, df = 4, p = 0.015), the overall ecology underscores the persistent burden of Enterobacterales in hospital-acquired critical care infections [23,24].

Implementation of the multidisciplinary AMSP significantly enhanced clinical protocol compliance and rationalized bedside prescribing decisions [14, 26]. Among culture-positive episodes, susceptibility-guided antibiotic escalation increased from 24.0% (362/1,507) to 53.0% (985/1,858) (p < 0.001), ensuring timely, targeted coverage for resistant isolates and preventing clinical failure [24,27]. Culture-directed de-escalation accounted for 21.6% (402/1,858) post-implementation compared with 56.9% (858/1,507) at baseline (p < 0.001), while appropriate continuation of empirical regimens without modification increased from 19.0% (287/1,507) to 25.3% (471/1,858) (p < 0.001) [26,28]. This therapeutic distribution reflects two interdependent clinical mechanisms. First, institutional empirical prescribing adherence improved markedly from 46.0% to 72.1% (p < 0.001); because initial empirical choices were already tailored to departmental syndromic guidelines and our hospital antibiograms, fewer patients received inappropriately broad upfront coverage requiring step-down. Second, enhanced pre-antibiotic diagnostic sampling unmasked true baseline multidrug-resistant pathogens (notably ESBL-producing and carbapenem-resistant Enterobacterales) that appropriately prompted susceptibility-guided escalation to definitive therapy [24,27].

Consequently, population-level reductions in drug pressure were achieved not by post-culture de-escalation alone, but through timely discontinuation in culture-negative or clinically stable patients (where appropriate continuation rose from 38.5% to 66.8%) and strict enforcement of surgical prophylaxis stop-orders [14, 16]. Adherence to standard SAP protocols improved substantially from 35.5% (1,352/3,812) to 63.2% (2,083/3,296; χ² = 544.00, p < 0.001), curbing routine postoperative antimicrobial prolongation beyond 24 hours [29, 30]. Prolonged postoperative broad-spectrum prophylaxis remains common across Indian surgical centres due to concerns over surgical site infections in high-volume settings [14, 29]. Multidisciplinary audit and clinical feedback helped align surgical prescribing with evidence-based recommendations, although residual non-compliance (36.8%) underscores the need for automated electronic stop-orders [26,29,30].

Longitudinal monthly tracking demonstrated a marked, sustained reduction in cumulative antimicrobial drug pressure following AMSP rollout [24, 31]. Overall consumption density decreased by 44.0% (120.04 to 67.17 DDD/100 bed-days; p < 0.001), while therapeutic duration density fell by 39.6% (981.23 to 592.91 DOT/1,000 bed-days; p < 0.001) [24, 31]. Segmented regression modelling confirmed that these reductions represented immediate, statistically significant level step-changes (β2) directly attributable to the stewardship rollout rather than pre-existing secular trends [32]. Meropenem exposure remained strictly controlled and unchanged between phases (4.7% vs. 5.0%; p = 0.549), corresponding to a very low post-implementation consumption density of 0.92 DDD/100 bed-days and 18.41 DOT/1,000 bed-days [16,24]. This carbapenem-sparing approach was supported by a managed increase in carbapenem-sparing β-lactam/β-lactamase inhibitor combinations, specifically piperacillin-tazobactam (23.0% to 28.4%; p < 0.001) and cefoperazone-sulbactam (45.0% to 48.7%; p = 0.001), alongside a reduction in fluoroquinolone exposure (ciprofloxacin: 46.3% to 44.0%; p = 0.044) [27,33]. Recent Indian studies support this carbapenem-sparing strategy, with Chagalamari et al. reporting piperacillin-tazobactam susceptibility in 76.9% of bloodstream isolates from critically ill ICU patients [34]. Furthermore, modest increases in the prescribing of oral step-down agents such as amoxicillin-clavulanic acid (30.3% to 36.0%; p < 0.001), metronidazole (29.0% to 32.5%; p = 0.001), and cotrimoxazole (12.0% to 14.6%; p = 0.001) reflect active stewardship initiatives prioritizing timely IV-to-oral switch protocols once clinical stabilization occurred [33].

Limitations

This study has several important methodological limitations that warrant consideration. First, although its single-centre retrospective observational design without a concurrent control group cannot rule out all concurrent institutional initiatives, the application of interrupted time-series segmented regression helped control for pre-intervention secular trends and quantify the immediate intervention effect [14,18,32]. However, the absence of a concurrent control group means that secular trends, seasonal variation, and concurrent infection control measures cannot be fully excluded as contributors to the observed changes. Second, patient-level clinical safety outcomes, including 30-day all-cause mortality, ICU length of stay, *Clostridioides difficile* infection rates, and individualized multidrug-resistant organism acquisition, were not captured in this operational pharmacy and microbiology database, precluding direct correlation between reduced antibiotic pressure and clinical patient outcomes [26,27]. Third, unadjusted case-mix differences (notably the shift toward higher SICU admissions and a higher proportion of male patients post-implementation) could not be controlled via multivariate regression because standardized physiological illness severity scores (such as APACHE II or SOFA) were not routinely recorded in the hospital database [3,19]. Fourth, the interrupted time series included only 17 monthly data points, limiting the precision of slope estimates. Fifth, residual and time-varying confounding (e.g., seasonal variation, changes in surgical case complexity, and staff turnover) could not be fully accounted for. Sixth, changes in diagnostic ascertainment due to improved EMR documentation may account for part of the gains in process measures [17,20]. Seventh, the role of other healthcare-associated infections (e.g., central line-associated bloodstream infection (CLABSI), catheter-associated urinary tract infection (CAUTI), and ventilator-associated pneumonia (VAP)) was not systematically captured, and concurrent infection control measures may have contributed to the observed reductions in antimicrobial consumption [14,21]. Finally, the single-centre design limits generalizability to other settings with different case mixes, resistance patterns, or stewardship infrastructure.

## Conclusions

In conclusion, the implementation of a multidisciplinary AMSP in an Indian tertiary critical care setting was associated with significant and sustained reductions in total antimicrobial consumption and duration density, alongside marked improvements in pre-antibiotic diagnostic sampling and adherence to empirical prescribing guidelines. These findings support a strong temporal association between structured stewardship interventions and meaningful improvements in rational drug utilization and diagnostic practices in resource-limited intensive care environments without compromising carbapenem-sparing protocols. However, the observational pre-post design cannot establish causality, and prospective studies with concurrent controls and clinical endpoints are needed to confirm these findings.
